# Supplementary material for: The Feasibility of Cervical Elastography in Predicting Preterm Delivery in Singleton Pregnancy with Short Cervix Following Progesterone Treatment
Source: Int J Environ Res Public Health. 2021 Feb 19;18(4):2026. doi: 10.3390/ijerph18042026 (PMC7922916; doi:10.3390/ijerph18042026)
Supplement: Supplementary file 1 [file ijerph-18-02026-s001.pdf]

Supplementary Materials

Table S1. Comparison of cervical length and elastographic parameters measured at pre- and post-progesterone treatment.

variable	Total			Control			sPTD		
	PP0 (n = 114)	PP1 (n = 97)	*p*-value	PP0 (n = 106)	PP1 (n = 91)	*p*-value	PP0 (n = 9)	PP1 (n = 6)	*p*-value
IOS	0.21 (0.17–0.28)	0.22 (0.17–0.29)	0.469	0.20 (0.17–0.27)	0.21 (0.17–0.28)	0.468	0.28 (0.25–0.34)	0.34 (0.30–0.40)	0.409
EOS	0.25 (0.20–0.34)	0.26 (0.22–0.33)	0.612	0.24 (0.19–0.34)	0.25 (0.21–0.32)	0.601	0.29 (0.28–0.38)	0.33 (0.29–0.40)	0.553
ECI	3.16 (2.45–,3.93)	3.03 (2.37–3.97)	0.750	3.13 (2.21–3.87)	2.98 (2.34–3.93)	0.723	3.62 (2.90–4.47)	4.10 (3.92–4.22)	0.776
HR	73.65 (62.39–82.79)	73.19 (63.55–84.25)	0.696	78.28 (62.43–83.48)	74.41 (65.77–84.59)	0.708	68.44 (53.76–70.39)	56.30 (41.34–64.19)	0.272
Cervical length	22.00 (20.05–24.00)	24.00 (21.00–26.00)	<0.001	22.00 (20.42–24.00)	24.00 (21.00–26.00)	<0.001	20.50 (13.90–23.25)	24.00 (20.70–27.83)	0.300

ECI, elasticity contrast index; EOS, mean of external os strain; HR, hardness ratio; IOS, mean of internal os strain; PP0, baseline; PP1, post-treatment 1 week. Data are presented as the median (range).
